# Transcriptional profile of *P. syringae* pv. phaseolicola NPS3121 at low temperature: Physiology of phytopathogenic bacteria

**DOI:** 10.1186/1471-2180-13-81

**Published:** 2013-04-12

**Authors:** Jackeline Lizzeta Arvizu-Gómez, Alejandro Hernández-Morales, Juan Ramiro Pacheco Aguilar, Ariel Álvarez-Morales

**Affiliations:** 1 Departamento de Ingeniería Genética, CINVESTAV-IPN Unidad Irapuato, Apdo Postal 629, Irapuato, Gto, CP 36821, Mexico; 2 Unidad Académica Multidisciplinaria Zona Huasteca, Universidad Autónoma de San Luis Potosí, Romualdo del Campo 501, Fraccionamiento Rafael Curiel, Cd. Valles, San Luis Potosí, CP 79060, Mexico; 3 Laboratorio de Plantas y Biotecnología Agrícola. Facultad de Química, Universidad Autónoma de Querétaro, Cerro de las campanas S/N, CU. Col. Las Campanas, Querétaro Qro, CP 76010, Mexico

**Keywords:** *P. syringae* pv. phaseolicola, Phytopathogen, Low temperature, Transcriptional profile, Physiology

## Abstract

**Background:**

Low temperatures play key roles in the development of most plant diseases, mainly because of their influence on the expression of various virulence factors in phytopathogenic bacteria. Thus far, studies regarding this environmental parameter have focused on specific themes and little is known about phytopathogenic bacteria physiology under these conditions. To obtain a global view regarding phytopathogenic bacteria strategies in response to physiologically relevant temperature changes, we used DNA microarray technology to compare the gene expression profile of the model bacterial pathogen *P. syringae* pv. phaseolicola NPS3121 grown at 18°C and 28°C.

**Results:**

A total of 236 differentially regulated genes were identified, of which 133 were up-regulated and 103 were down-regulated at 18°C compared to 28°C. The majority of these genes are involved in pathogenicity and virulence processes. In general, the results of this study suggest that the expression profile obtained may be related to the fact that low temperatures induce oxidative stress in bacterial cells, which in turn influences the expression of iron metabolism genes. The expression also appears to be correlated with the profile expression obtained in genes related to motility, biofilm production, and the type III secretion system.

**Conclusions:**

From the data obtained in this study, we can begin to understand the strategies used by this phytopathogen during low temperature growth, which can occur in host interactions and disease development.

## Background

During their life cycles, phytopathogenic bacteria possess an epiphyte growth stage during which they can grow and reproduce on the surface of a plant without causing disease. However, when conditions are favorable, the bacteria enter a pathogenic stage that leads to disease development. It is known that a complex interaction between key factors must exist for the development of the disease in plants, represented as “disease triangle”. This involves the interaction of a susceptible host, a virulent pathogen, and environmental conditions favorable for disease development [1,2]. Regarding environmental conditions, temperature is a key factor in most plant diseases, which are favored mainly by low temperatures [1,3,4]. The influence of low temperature on disease development is predominantly due to the expression of various pathogenicity and/or virulence factors by the pathogens, which influences plant health. Several bacterial phytopathogens, such as *Pseudomonas syringae* and *Erwinia sp*., produce disease in their host plants in response to low temperature, which appears to be the cue for these phytopathogens to produce virulence factors, including toxins, cell-wall degrading enzymes, and effector proteins [4]. Thus, low temperatures are an important environmental parameter in the development of most diseases in plants.

One of the most common and economically important diseases is the bean disease (*Phaseolus vulgaris* L.) known as “halo blight” because it causes major field crop losses. This disease, caused by the bacterial pathogen *P. syringae* pv. phaseolicola, affects both the leaves and pods [5-7]. Cool temperatures (less than 25°C) favor disease development, a condition that also favors production of the major virulence factor of the pathogen, known as phaseolotoxin [8,9]. Phaseolotoxin is a non-host specific and chlorosis-inducing toxin that acts as a reversible inhibitor of the enzyme ornithine carbamoyltransferase (OCTase; EC2.1.3.3), which catalyzes the conversion of ornithine to citruline in the arginine biosynthetic pathway [10,11]. The production of phaseolotoxin by *P. syringae* pv. phaseolicola is regulated mainly by temperature and is optimally produced at 18°C-20°C, whereas at 28°C (the optimal growth temperature for this bacterium), the toxin is not detected [8,9]. Genes whose products are involved in phaseolotoxin synthesis are clustered within of a chromosomal region known as the “Pht cluster”, whose expression is also low temperature (18°C) dependent [12]. Thus, like other phytopathogenic bacteria, low temperatures play an important role in *P. syringae* pv. phaseolicola for the development of halo blight disease. However, research related to this environmental parameter has focused only on phaseolotoxin production and little is known about the bacterium physiology under these conditions. Likewise, studies performed in other phytopathogenic bacteria have focused on specific topics regarding low temperature function [4].

Global knowledge about the strategies used by these phytopathogens, in terms of temperature change which influences virulence stage and disease development, is very scarce and most of these studies have focused on animal pathogens where high temperature caused this effect [13,14]. Therefore, this study was undertaken with the objective to understand how phytopathogenic bacteria, in particular the bacterial pathogen *P. syringae* pv. phaseolicola NPS3121, respond to temperature changes related to the development of the most of plant diseases.

## Results and discussion

### Low temperature (18°C) negatively affects the growth rate of *P. syringae* pv. phaseolicola NPS3121

**Figure 1:**
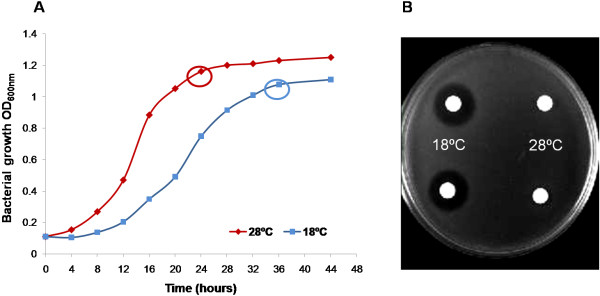
**Low temperature decreases the bacterial growth rate and favors phaseolotoxin production.** Panel **A** shows the bacterial growth curves of *P. syringae* pv. phaseolicola NPS3121 grown at 18°C and 28°C. The low temperature decreases the growth rate of the bacterium and favors phaseolotoxin production (panel **B**). The circles indicate the growth stage in which the RNA extraction was performed.

To obtain a global view regarding the strategies used by *P. syringae* pv. phaseolicola NPS3121 in response to physiologically relevant temperature changes, we used DNA microarray technology. We compared gene expression profiles in the *P. syringae* pv. phaseolicola NPS3121 wild-type (wt) strain grown at 18°C and 28°C in M9 minimal media. These temperatures represent conditions that either favor the development of the disease (18°C) or do not (28°C) [8]. Initially, to evaluate the effect of temperature and establish the growth stage for this study, we performed bacterial growth curves of the *P. syringae* pv. phaseolicola NPS3121 strain grown under the conditions mentioned above. The results showed that at low temperature (18°C), the growth rate of the bacteria decreases approximately 0.5-fold relative to 28°C (Figure 1A). This behavior was reproducible in all performed kinetics. The effect of low temperature on the growth rate of several *Pseudomonas syringae* strains, including pv. phaseolicola, had been previously observed with similar results to this study [15]. Because previous results from our group indicated that during the transition phase, low temperature-induced differential expression in the phaseolotoxin synthesis genes (Pht cluster) occurs [12], we performed this study with cells harvested during this growth stage, which allowed us to use this cluster as a control for the microarray and ensure the virulent stage of the bacterium. Thus, parallel cultures of *P. syringae* pv. phaseolicola NPS3121 grown at 28°C and 18°C were harvested at the transition phase and RNA was extracted. The results presented in this work reflect the adapted state and significant genes, whose expression is differentially maintained over long-term growth at a given temperature.

### Differentially expressed genes at 18°C are distributed throughout the chromosome and comprise several functional categories

**Figure 2:**
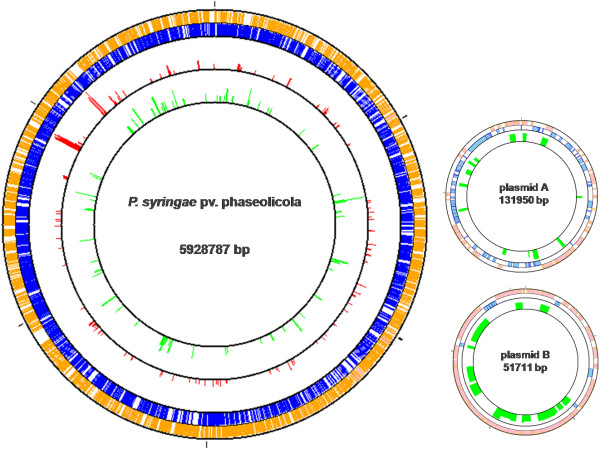
**Distribution and location of differentially expressed genes at 18°C in the *P. syringae *pv. phaseolicola genome.** Differentially regulated genes were analyzed using the GenoMap software and their distribution and location in the bacterium genome was determined. The red bars depict the distribution of up-regulated genes and the green bars represent the down-regulated genes at 18°C.

The differentially expressed genes were identified using a cut-off criteria of ≥1.5 for up-regulated and ≤0.6 for down-regulated genes (p-value ≤ 0.05). A total of 236 differentially regulated genes were identified, of which 133 were up-regulated and 103 were down-regulated at 18°C relative to 28°C. Analyses about the distribution and location of the genes in the *P. syringae* pv. phaseolicola 1448A sequenced genome, showed that the differentially expressed genes at 18°C are not located in a single chromosomal region of *P. syringae* pv. phaseolicola, but rather are distributed throughout the genome. Furthermore, only down-regulated genes were distributed in both plasmids of this strain (Figure 2). This pattern of distribution had been observed in preliminary assays, in which a Tn5-derived promoter probe was used to search for genes whose expression was temperature dependent; however, the authors reported the location of only a few genes throughout the genome [16].

For the purposes of this study, the differentially regulated genes were analyzed and manually grouped into categories based on their putative role in biological processes (Tables 1 and 2). In general, data analyses show that the majority of the differentially regulated genes relate to the pathogenicity and/or virulence process of the bacterium.

**Table 1:** **Genes up-regulated at 18°C in *P. syringae *pv. phaseolicola NPS3121**

**Gen/ORF**	**Gene product**	**Ratio**
**Cluster 1: Phaseolotoxin production (Pht cluster)**		
PSPPH_4299	Hypothetical protein (*phtU)*	11.86
PSPPH_4300	Membrane protein, putative *(phtT)*	8.70
PSPPH_4301	Adenylylsulfate kinase *(phtS)*	13.50
PSPPH_4302	Conserved hypothetical protein *(phtQ)*	6.23
PSPPH_4305	Hypothetical protein *(phtO)*	8.78
PSPPH_4306	Hypothetical protein *(phtM)*	15.90
PSPPH_4306	Hypothetical protein *(phtM)*	7.29
PSPPH_4307	pyruvate phosphate dikinase PEP/pyruvate binding subunit	23.74
PSPPH_4317	Hypothetical protein	11.52
PSPPH_4323	Hypothetical protein	2.13
*argK*	control	3.30
*phtA*	control	4.96
*phtD*	control	6.50
*desI*	control	14.97
*phtL*	control	7.64
*phtMN*	control	1.81
*amtA*	control	10.34
**Cluster 2: Genes involved in Non-ribosomal synthesis**		
PSPPH_4538	transposon Tn7-like transposase protein A	1.67
PSPPH_4539	transposon Tn7-like transposase protein B	1.70
PSPPH_4544	hypothetical protein PSPPH_4544	8.08
PSPPH_4544	hypothetical protein PSPPH_4544	7.26
PSPPH_4546	hypothetical protein	9.44
PSPPH_4549	hypothetical protein PSPPH_4549	9.13
PSPPH_4553	major facilitator family protein	15.83
PSPPH_4554	arginine aminomutase, putative	12.67
PSPPH_4555	conserved hypothetical protein	7.46
**Cluster 3: Type VI secretion system**		
PSPPH_0122	hcp	2.13
PSPPH_0124	hypothetical protein	1.66
PSPPH_0125	icmF	1.94
PSPPH_0131	HsiG	1.61
PSPPH_0135	hypothetical protein	1.64
PSPPH_4978	prophage PSPPH06, putative reverse transcriptase/maturase	1.65
PSPPH_4979	prophage PSPPH06, putative reverse transcriptase/maturase	2.68
PSPPH_4984	prophage PSPPH06, site-specific recombinase, phage integrase family	1.70
**Cluster 4: Genes involved in membrane synthesis**		
PSPPH_1430	leucine-rich repeat domain protein	1.75
PSPPH_1464	lipoprotein, putative	1.66
PSPPH_1708	ABC transporter, periplasmic substrate-binding protein	1.84
PSPPH_2260	UTP-glucose-1-phosphate uridylyltransferase	1.72
PSPPH_2542	membrane protein, putative	1.94
PSPPH_2643	outer membrane efflux protein	1.68
PSPPH_2654	lipoprotein, putative	1.55
PSPPH_2842	lipoprotein, putative	1.55
PSPPH_3226	glycosyl transferase, group 1 family protein	1.72
PSPPH_3288	predicted periplasmic lipoprotein	2.05
PSPPH_3810	lipoprotein	1.71
PSPPH_3916	membrane protein, putative	1.86
PSPPH_4139	UDP-N-acetylglucosamine 1-carboxyvinyltransferase	1.55
PSPPH_4669	acetyltransferase, GNAT family	1.73
PSPPH_4682	lipopolysaccharide biosynthesis protein, putative	3.03
PSPPH_5220	inner membrane protein, 60 kDa	1.61
**Cluster 5: Genes involved in motility**		
PSPPH_0730	type IV pilus-associated protein, putative	1.74
PSPPH_0818	type IV pilus prepilin peptidase PilD	1.53
PSPPH_0820	type IV pilus biogenesis protein PilB	1.53
PSPPH_1200	pili assembly chaperone	2.03
PSPPH_3387	flagellar regulator FleQ	1.64
PSPPH_3880	CheW domain protein WspB	1.94
PSPPH_3881	methyl-accepting chemotaxis protein WspA	1.5
**Cluster 6: Oxidative stress response genes and iron metabolism**		
PSPPH_1309	cysteine desulfurase IscS	1.93
PSPPH_1311	iron-sulfur cluster assembly protein IscA	1.69
PSPPH_1909	RNA polymerase sigma-70 family protein. pvdS	1.59
PSPPH_1923	pyoverdine sidechain peptide synthetase I, epsilon-Lys module	1.70
PSPPH_2117	FecR protein superfamily	2.05
PSPPH_3007	iron ABC transporter, permease protein, putative	1.61
PSPPH_3274	catalase KatB	2.31
PSPPH_3274	catalase KatB	1.65
PSPPH_3753	siderophore biosynthesis protein	1.57
**Cluster 7: Unknown function**		
PSPPH_0317	conserved hypothetical protein	1.81
PSPPH_0611	conserved hypothetical protein	2.15
PSPPH_0612	hypothetical protein PSPPH_0612	1.94
PSPPH_1142	hypothetical protein PSPPH_1142	1.58
PSPPH_1230	hypothetical protein PSPPH_1230	1.59
PSPPH_1243	conserved hypothetical protein	1.98
PSPPH_1637	hypothetical protein	2.25
PSPPH_1835	conserved hypothetical protein	1.72
PSPPH_1938	conserved hypothetical protein	1.52
PSPPH_2103	conserved hypothetical protein	1.89
PSPPH_2116	conserved hypothetical protein	1.58
PSPPH_2147	hypothetical protein PSPPH_2147	2.51
PSPPH_2578	conserved hypothetical protein	1.53
PSPPH_3212	conserved hypothetical protein	1.53
PSPPH_3262	conserved hypothetical protein	1.60
PSPPH_5014	conserved hypothetical protein	1.52
**Cluster 8: Uncharacterized Function**		
PSPPH_0210	DNA repair protein RadC	1.56
PSPPH_0398	glutamate synthase, large subunit	2.63
PSPPH_0581	radical SAM domain protein	1.53
PSPPH_0620	DNA primase	2.48
PSPPH_0622	O-sialoglycoprotein endopeptidase	1.87
PSPPH_0625	2-amino-4-hydroxy-6-hydroxymethyldihydropteridine pyrophosphokinase	1.62
PSPPH_0627	SpoVR like family protein	2.10
PSPPH_0629	protein kinase	1.65
PSPPH_0703	phosphonate ABC transporter permease protein phnE	1.73
PSPPH_1141	ISPsy20, transposase IstB	1.51
PSPPH_1150	conserved domain protein-Divergente HU family	1.61
PSPPH_1179	DNA-binding response regulator GltR	1.54
PSPPH_1244	transcriptional regulator, AsnC family	1.89
PSPPH_1306	RNA methyltransferase, TrmH family, group 1	1.545
PSPPH_1378	Methionyl-tRNA synthetase (Methionine--tRNA ligase)(MetRS)	2.56
PSPPH_1406	ATP-dependent helicase, DinG family	1.77
PSPPH_1468	nucleic acid binding protein	1.58
PSPPH_1595	transcriptional regulator, GntR family	2.58
PSPPH_1661	cvpA family protein	1.66
PSPPH_1746	oxidoreductase, aldo/keto reductase family	1.92
PSPPH_2216	zinc carboxypeptidase domain protein	1.89
PSPPH_2221	precorrin-4 C11-methyltransferase	1.52
PSPPH_2506	L-arabinose ABC transporter, periplasmic L-arabinose-binding protein	1.62
PSPPH_2551	oxidoreductase, putative	1.84
PSPPH_2563	transcriptional regulator, GntR family	1.53
PSPPH_2580	transcriptional regulator, LysR family	1.97
PSPPH_2620	5-methyltetrahydrofolate--homocysteine methyltransferase	1.85
PSPPH_2690	oxidoreductase, FAD-binding, putative	1.56
PSPPH_2781	TspO/MBR family protein	1.99
PSPPH_2840	sodium/hydrogen exchanger family protein	1.55
PSPPH_2847	general secretion pathway protein GspK, putative	1.89
PSPPH_3045	transporter, AcrB/AcrD/AcrF family	1.64
PSPPH_3252	glycolate oxidase, GlcD subunit	1.97
PSPPH_3291	oxidoreductase, molybdopterin-binding	1.88
PSPPH_3294	DNA-binding heavy metal response regulator	1.81
PSPPH_3654	transcriptional regulator, TetR family	1.51
PSPPH_3906	sensor histidine kinase	1.65
PSPPH_3946	DNA repair protein RecO	1.66
PSPPH_3962	DNA-binding response regulator TctD	1.77
PSPPH_4137	histidinol dehydrogenase	1.63
PSPPH_4151	RNA polymerase sigma-54 factor RpoN	1.69
PSPPH_4152	ribosomal subunit interface protein	1.86
PSPPH_4332	DNA repair protein RadA	1.76
PSPPH_4372	RNA 2'-phosphotransferase	1.55
PSPPH_4634	bmp family protein	2.99
PSPPH_4641	YccA	1.68
PSPPH_4717	dethiobiotin synthetase	2.09
PSPPH_4866	proline-specific permease proY	1.54
PSPPH_4925	imidazole glycerol phosphate synthase, glutamine amidotransferase subunit	1.62
PSPPH_5142	oxaloacetate decarboxylase alpha subunit	2.35

The described functions were obtained from the literature. The up-regulated genes were identified using cutoff criteria ≥1.5 of ratio. The ratio is in relation to expression levels obtained between 18°C and 28°C (18°C/28°C). Control: corresponds to genes obtained by PCR amplification that were printed in the microarray.

**Table 2:** **Genes down-regulated at 18°C in *P. syringae *pv. phaseolicola NPS3121**

**Gen/ORF**	**Gene product**	**Ratio**
**Cluster 9: Alginate synthesis**		
PSPPH_1112	alginate biosynthesis protein AlgX	0.52
PSPPH_1113	alginate biosynthesis protein AlgG	0.19
PSPPH_1114	alginate biosynthesis protein AlgE	0.18
PSPPH_1115	alginate biosynthesis protein AlgK	0.19
PSPPH_1118	alginate biosynthesis protein AlgD	0.46
PSPPH_1119	conserved hypothetical protein	0.46
*algD*	*algD* (control)	0.25
**Cluster 10: Plant-Pathogen interactions**		
PSPPH_A0075	type III effector HopW1-2, truncated	0.60
PSPPH_A0127	type III effector HopAB1	0.42
PSPPH_A0127	type III effector HopAB1	0.65
PSPPH_A0127	virA type III HopAB1 (control)	0.57
PSPPH_A0120	avrC type III effector AvrB2 (control)	0.53
PSPPH_A0010	avrD type III effector hopD1 (control)	0.56
PSPPH_3992	pectin lyase	0.62
PSPPH_3993	acetyltransferase, GNAT family	0.57
PSPPH_A0072	polygalacturonase	0.50
**Cluster 11: Type IV secretion system**		
PSPPH_B0022	transcriptional regulator, PbsX family	0.65
PSPPH_ B0023	transcriptional regulator	0.64
PSPPH_ B0025	conjugal transfer protein	0.65
PSPPH_ B0027	conjugal transfer protein	0.65
PSPPH_ B0028	conjugal transfer protein	0.61
PSPPH_ B0031	conjugal transfer protein	0.65
PSPPH_ B0032	conjugal transfer protein	0.61
PSPPH_ B0034	conjugal transfer protein	0.62
PSPPH_ B0035	conjugal transfer protein	0.66
PSPPH_ B0036	conjugal transfer protein	0.51
PSPPH_ B0041	conjugal transfer protein	0.58
**Cluster 12: Heat-shock proteins**		
PSPPH_0381	heat shock protein HslVU, ATPase subunit HslU	0.65
PSPPH_0742	clpB protein	0.54
PSPPH_4077	chaperonin, 60 kDa. groEL	0.29
PSPPH_4206	dnaK protein	0.28
PSPPH_4206	dnaK protein	0.57
PSPPH_4207	heat shock protein GrpE	0.65
**Cluster 13: Genes related with nucleic acids synthesis**		
PSPPH_4598	DNA-directed RNA polymerase, beta' subunit	0.59
PSPPH_4599	DNA-directed RNA polymerase, beta' subunit	0.57
PSPPH_2495	DNA polymerase II	0.57
PSPPH_B0043	DNA topoisomerase III	0.64
PSPPH_A0002	Replication protein	0.54
**Cluster 14: Unknown function**		
PSPPH_0220	conserved hypothetical protein	0.64
PSPPH_0609	hypothetical protein PSPPH_0609	0.54
PSPPH_2482	conserved hypothetical protein	0.63
PSPPH_2855	hypothetical protein PSPPH_2855	0.43
PSPPH_3333	conserved hypothetical protein	0.36
PSPPH_3625	conserved hypothetical protein	0.59
PSPPH_4047	conserved hypothetical protein	0.66
PSPPH_A0040	hypothetical protein PSPPH_A0040	0.66
PSPPH_B0048	conserved hypothetical protein	0.60
**Cluster 15: Uncharacterized function**		
PSPPH_0012	glycyl-tRNA synthetase, alpha subunit	0.63
PSPPH_0033	3-oxoadipate enol-lactonase, putative	0.65
PSPPH_0072	membrane protein, putative	0.63
PSPPH_0080	ATP-dependent DNA helicase Rep	0.43
PSPPH_0117	phospholipase D family protein	0.63
PSPPH_0215	aldehyde dehydrogenase family protein	0.35
PSPPH_0296	colicin/pyocin immunity family protein	0.58
PSPPH_0360	periplasmic glucan biosynthesis protein	0.63
PSPPH_1072	oxidoreductase, short chain dehydrogenase/reductase family	0.57
PSPPH_1181	glucose ABC transporter, periplasmic glucose-binding protein, putative	0.65
PSPPH_1211	cytochrome o ubiquinol oxidase, subunit I	0.55
PSPPH_1508	acetyltransferase, GNAT family	0.35
PSPPH_1518	ATP-dependent DNA helicase RecQ	0.53
PSPPH_1575	CAIB/BAIF family protein	0.65
PSPPH_1759	plasmid stabilization system family protein	0.53
PSPPH_1762	transcriptional regulator, AsnC family	0.54
PSPPH_1917	cation ABC transporter, periplasmic cation-binding protein	0.60
PSPPH_1921	peptidase	0.58
PSPPH_1963	electron transfer flavoprotein-ubiquinone oxidoreductase, putative	0.38
PSPPH_2053	membrane protein, putative	0.65
PSPPH_2057	2-methylcitrate synthase	0.62
PSPPH_2159	dehydrogenase, isocitrate/isopropylmalate family	0.60
PSPPH_2246	4-alpha-glucanotransferase	0.66
PSPPH_2695	peptide ABC transporter, permease protein	0.45
PSPPH_2868	major facilitator family transporter	0.63
PSPPH_2892	TonB-dependent siderophore receptor, putative	0.62
PSPPH_2897	yersiniabactin non-ribosomal peptide synthetase	0.40
PSPPH_2899	yersiniabactin polyketide/non-ribosomal peptide synthetase	0.58
PSPPH_2904	isochorismate synthase	0.55
PSPPH_3100	isocitrate dehydrogenase, NADP-dependent	0.63
PSPPH_3251	maleylacetoacetate isomerase	0.53
PSPPH_3528	acetate--CoA ligase	0.52
PSPPH_3558	aconitate hydratase 2	0.61
PSPPH_3782	porin D	0.42
PSPPH_3985	3-oxoacyl-[acyl-carrier protein] reductase	0.54
PSPPH_4221	unnamed protein product	0.44
PSPPH_4654	smtA protein	0.47
PSPPH_4703	coenzyme PQQ biosynthesis protein PqqF	0.32
PSPPH_4805	oxidoreductase FAD-binding domain/oxidoreductase NAD-binding domain/2Fe-2S iron-sulfur cluster binding domain protein	0.55
PSPPH_4833	Rhs family protein	0.33
PSPPH_4859	transporter, BCCT family	0.65
PSPPH_4869	cadmium-translocating P-type ATPase	0.54
PSPPH_4885	D-3-phosphoglycerate dehydrogenase	0.56
PSPPH_4938	amino acid ABC transporter, ATP-binding protein	0.61
PSPPH_4962	prophage PSPPH06, C4-type zinc finger protein, DksA/TraR family	0.35
PSPPH_5024	acetyltransferase, GNAT family	0.64
PSPPH_5027	acetyltransferase, GNAT family	0.64
PSPPH_5170	acyltransferase family protein	0.60
PSPPH_A0062	LysR-family transcription regulator SinR	0.45
PSPPH_A0083	IS801, transposase	0.64
PSPPH_A0109	sulfotransferase, putative	0.49
PSPPH_A0129	Yersinia/Haemophilus virulence surface antigen family	0.53
PSPPH_A0132	ISPsy16, transposase	0.66
PSPPH_A0145	conjugal transfer protein	0.56
PSPPH_B0004	RulB protein	0.63
PSPPH_B0050	relaxase, putative	0.65
PSPPH_B0059	exeA-like protein	0.64

The described functions were obtained from the literature. The down-regulated genes were identified using cutoff criteria ≤ 0.6 of ratio. The ratio is in relation to the expression levels obtained between 18°C and 28°C (18°C/28°C). Control: corresponds to genes obtained by PCR amplification that were printed in the microarray.

### Control and reliability of the microarray: Pht cluster genes and phaseolotoxin synthesis are induced at 18°C

**Figure 3:**
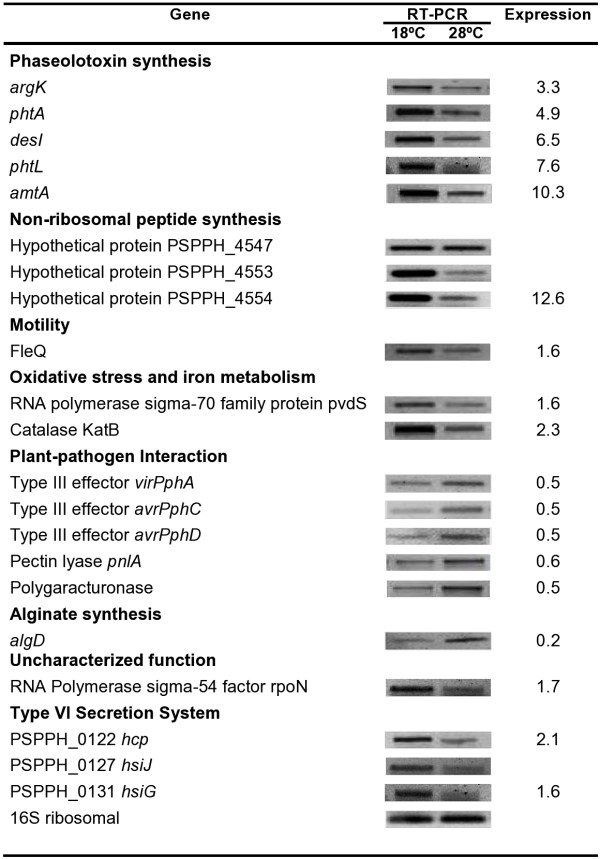
**Microarray validation using RT-PCR analyses.** RT-PCR validates the microarray results. ^a^ Corresponds to expression levels obtained in the microarray for these genes. The remaining genes do not show expression levels because they were not printed on the microarray.

As expected, the microarray data showed that the Pht cluster genes (Cluster 1), involved in phaseolotoxin synthesis, were up-regulated at 18°C relative 28°C, thus confirming the previously reported data [12]. Thirteen of the 23 genes that comprise the Pht region were highly expressed at 18°C relative 28°C, which was consistent with the conditions of phaseolotoxin synthesis observed in the growth inhibition assays (Figure 1B). Only 13 of the 23 Pht cluster genes were activated because only these genes are printed on our microarray. However, these genes represent the five transcriptional units that comprise the Pht region [12]. To validate the microarray data, one gene from each transcriptional unit was selected for validation of their expression pattern by RT-PCR analyses (Figure 3). The variability in Pht cluster gene expression levels observed could suggest different regulation mechanisms for each of them. Thus far, it is known that there is transcriptional regulation for this group of genes mediated by temperature and only IHF protein has been identified as directly involved in the regulation of some of them [12,17]. The results regarding the Pht cluster can also be used as control of the microarray, ensuring the reliability of the results obtained in this study.

### Genes involved in non-ribosomal peptide synthesis are induced at low temperature

Another group of genes that was up-regulated at 18°C in *P. syringae* pv. phaseolicola NPS3121 comprise Cluster 2, corresponding to genes involved in non-ribosomal peptide synthesis (NRPS) [18]. NRPS is an alternative pathway that allows production of polypeptides via a different mechanism than the traditional translation pathway. Peptides are created by enzymatic complexes called synthetases. Through NRPS, some bacteria produce several secondary metabolites, such as siderophores, antibiotics, or toxins that contribute to the fitness and/or pathogenicity of the bacterium [19,20]. In our microarray, six genes that encode four hypothetical proteins (PSPPH_4544, PSPPH_4546, PSPPH_4549, and PSPPH_4555), a facilitator family protein (PSPPH_4553), and an arginine aminomutase (PSPPH_4554) were highly up-regulated at 18°C. These genes are located in a 27 kbp fragment, which also encodes a polyketide synthetase domain protein (PSPPH_4547) and a non-ribosomal peptide synthetase (PSPPH_4550). This region is delimited by genes encoding for transposases (PSPPH_4538 and PSPPH_4559), which were also induced in our microarray (Table 1, Cluster 2). Of all the genes of this region, only six genes were printed on the microarray and all of these were induced in the conditions evaluated. RT-PCR analyses confirmed the profile expression and temperature dependence of PSPPH_4553 and PSPPH_4554, thus validating the microarrays results (Figure 3). The expression of at least one of these genes (PSPPH_4550) in temperature dependence had been previously observed with similar results [21]. In *P. syringae* pv. phaseolicola NPS3121, it has been suggested that NRPS genes are part of a genomic island (IG) acquired by horizontal transfer and is postulated to be involved in phaseolotoxin synthesis during peptide assembly. However, only the PSPPH_4550 gene has been demonstrated to have a role in this process [21]. Based on this hypothesis, the profile expression obtained for this group of genes at 18°C could be congruent with the differential expression of the Pht cluster genes and the conditions for phaseolotoxin synthesis. However, the RT-PCR results for the PSPPH_4547 gene showed that the expression of this gene is independent of temperature, presenting constitutive behavior at both temperatures (Figure 3). Knowledge regarding the role of this *P. syringae* pv. phaseolicola gene group is limited and experimental work is still necessary. Likewise, is necessary to evaluate whether there is a relationship between these genes and phaseolotoxin synthesis genes, as has been previously proposed, or whether these genes participate in different biological processes that contribute to the fitness of the bacterium in low temperatures.

### In *P. syringae* pv. phaseolicola NPS3121, the Type VI secretion system (T6SS) is regulated by temperature

Recently, a new secretion system has been recognized, called the Type VI secretion system (T6SS). This system is encoded within the genomes of most Gram negative bacteria, including plant, animal, and human pathogens, as well as environmental strains. The T6SS components are usually encoded by a gene cluster that is thought to form a genomic island whose composition and number varies among species [22-24]. The *in silico* analyses have revealed that the genome of *P. syringe* pv. phaseolicola 1448A carries only one putative T6SS gene cluster (HSI) that comprises the region from PSPPH_0119 to PSPPH_0135. Furthermore, several genes putatively encoding some proteins of this system are scattered in the genome of this bacterium [24]. The microarrays results showed the induction of eight genes encoding proteins putatively involved in the T6SS in *P. syringe* pv. phaseolicola NPS3121 (Cluster 3). The PSPPH_0122 gene encodes a hemolysin-coregulated (Hcp) protein homolog, in addition to be an essential component of the secretion machinery, acts as an effector protein that is secreted through this system. The PSPPH_0124 gene encodes a hypothetical protein and the PSPPH_0125 gene encodes the IcmF protein, which in conjunction with the DotU protein (PSPPH_0126), act as associated structural proteins that anchor the secretion system in the cell membrane [25]. Within this cluster is also the PSPPH_0131 gene encoding the hsiG protein and the PSPPH_0135 gene that encodes a hypothetical protein. The expression of these genes at low temperature was confirmed by RT-PCR (Figure 3). Although PSPPH_ 4978, PSPPH_ 4979, and PSPPH_ 4984, which encode prophage PSPPH06 proteins, are not involved in T6SS, these genes were include within this group because their adjacent genes (PSPPH_ 4980 and PSPPH_ 4985) putatively encode Hcp proteins [24], which may be responsible for the induction levels obtained. This finding is being evaluated in our laboratory. The T6SS has been shown to play a key role in the virulence and pathogenesis of diverse bacterial pathogens, in some cases, by the secretion of effector proteins or toxins. However, its complete mechanism of action is poorly understood. The function of this system is not restricted to pathogenic processes because the T6SS also participates in other processes such as biofilm formation, stress sensing, symbiosis, root colonization, and nodule formation [26,27]. The role of the putative T6SS gene cluster in *P. syringae* pv. phaseolicola NPS3121 has not been evaluated so more experimental work is required. However, it has been demonstrated that T6SS in *P. syringae* pv. syringae B728a, which is phylogenetically identical to *P. syringae* pv. phaseolicola T6SS, it is not essential for leaf colonization and development of the disease [28]. Several reports have demonstrated that expression of the T6SS gene cluster is tightly regulated in different environmental conditions and low temperatures contribute to the expression of these genes in some pathogens [29]. This phenomenon is similar to our observation that low temperature (18°C) regulates T6SS genes expression. To our knowledge, this is the first report about expression of these genes of *P. syringae* pv. phaseolicola NPS3121 and the influence of low temperature on their expression.

### Cell envelope-associated changes are induced by low temperature

A universal response to low temperature includes changes in the lipid composition of membranes to help cope with the decrease in membrane fluidity caused by the cold. Microorganisms respond by increasing the unsaturated fatty acids level in membrane phospholipids, which helps to maintain membrane homeoviscosity so that its function is not affected. There are a variety of mechanisms that can alter membrane phospholipid composition in response to temperature change [30]. The conversion of saturated fatty acids into unsaturated fatty acids by desaturases enzymes is one of these pathways [30,31]. In our microarray and RT-PCR analyses (Figure 3, Cluster 1), the *desI* gene encoding a fatty acid desaturase was induced at 18°C, which might be involved in the unsaturation process, in a similar manner to the reported *desA* and *des* genes from *Synechosysteis* sp. PCC6803 and *Bacillus subtilis*, respectively. It has been observed that deletion of the *des* gene in *B. subtilis* produces a cold-sensitive phenotype and slower growth, thus demonstrating its role during adaptation to low temperatures [32]. In *P. syringae* pv. phaseolicola NPS3121, the *desI* gene is part of the *phtD* transcriptional unit of the Pht cluster (Cluster 1). Mutation of this gene produces a non-toxigenic phenotype relative to the wt strain. However, the relationship of *desI* with phaseolotoxin synthesis is still unknown [12]. Additionally, it has been observed that mutation in the *desI* gene decreases the growth rate at 18°C relative to the wt strain, suggesting a cold-sensitivity in the mutant strain (unpublished data).

Another of the mechanisms reported to be involved in membrane lipid composition changes correspond to *de novo* synthesis. The *fabF* and *lpxP* genes induced by low temperature participate in this process [33]. β-ketoacyl-ACP synthase II, the *fabF* gene product, converts palmitoleic acid to *cis*-vaccenic acid, which is in turn transferred by an acyltransferase (LpxP) into lipid A, a component of polysaccharides [33,34]. Although these two genes were not found in our microarray, several genes involved in cell wall biogenesis and membrane synthesis were identified (Cluster 4). These include the *murA* gene (PSPPH_4139) that is involved in peptidoglycan synthesis (a major component of cell wall), the PSPPH_4682 gene involved in polysaccharide synthesis, as well as three genes PSPPH_4669, PSPPH_3226, and *galU* (PSPPH_2260) that encode an acetyl-, glycosyl- and uridyl- transferase, respectively, which are likely associated with the transfer of these groups during polysaccharides synthesis. Additionally, it has been demonstrated that during cell envelope biogenesis, there is an increase in outer membrane lipoproteins, which increase connections with the cell wall [34,35]. In our analyses four genes (PSPPH_ 1464, PSPPH_2654, PSPPH_2842, and PSPPH_3810) encoding lipoproteins were induced, which may be related to outer membrane synthesis. The microarray results suggest that membrane component synthesis is activated in the conditions of our study and these changes are likely related to cell envelope remodeling to adapt to low temperatures.

### Low temperature induces expression of motility genes in *P. syringae* pv. phaseolicola NPS3121

Cluster 5 comprises genes induced at 18°C that are involved in bacterium motility. The data suggest that chemotaxis and rotation of flagella processes function in low temperatures on *P. syringae* pv. phaseolicola NPS3121. Two genes, PSPPH_3880 that encodes the membrane-bound methyl accepting chemotaxis protein (MCP)-like receptor WspA, and PSPPH_3881, that encodes the CheW-like scaffolding protein WspB, showed high transcripts levels at 18°C relative 28°C (Table 1). WspA and WspB are related to the chemotaxis process. Chemotaxis, as well as other types of taxis (e.g., thermotaxis), enables bacteria to approach beneficial environments and escape from hostile ones. Depending on the parameter monitored, bacteria will respond by either swimming toward attractants or retreating from repellants. Thus, the signal sensed by chemotaxis causes changes in flagellum motility [36]. Flagella gene expression is activated by the FleQ protein, which appears to be the highest regulator in the hierarchical flagellar biogenesis regulatory cascade in *P. aeruginosa*[37]. FleQ (PSPPH_3387) was induced in our study at 18°C and its expression was validated by RT-PCR (Figure 3), suggesting that the motility of *P. syringae* pv. phaseolicola NPS3121 is favored under this condition. Furthermore, four genes related to pili formation, which is also involved in bacterial movement, were induced at low temperature: PSPPH_0730 that encodes type IV pilus-associated protein, PSPPH_1200 that encodes a pili assembly chaperone, PSPPH_0818 that encodes PilD protein, and PSPPH_0820 that encodes PilB protein. Each of these genes has been associated with *P. syringae* pv. phaseolicola virulence because of their role in adhesion to the surface of host plants to initiate infection [38]. It has been reported that RpoN sigma factor regulates the expression of genes required for pili and flagella biosynthesis in *P. aeruginosa*[37,39]*.* Our microarray data and RT-PCR assays showed that the PSPPH_4151 gene (Cluster 8), which encodes the RpoN protein, was induced at 18°C, suggesting a similar regulation may occur in our strain (Figure 3). The results obtained suggest that *P. syringae* pv. phaseolicola NPS3121 motility is regulated by temperature, similar to those observed in the pathogens *Helicobacter pylori* and *E. coli*, whose motility patterns are altered by temperature changes [33,40].

**Figure 4:**
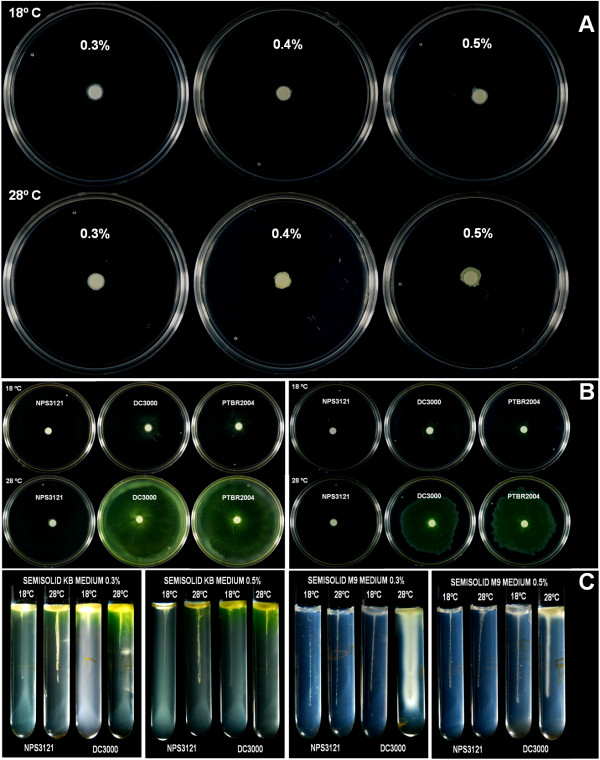
**Motility Tests of the *P. syringae *pv. phaseolicola NPS3121 strain.** (**A**) Motility assays performed in semisolid M9 media containing 0.3%, 0.4%, and 0.5% agar at 18°C and 28°C. (**B**) Motility assays in semisolid KB media containing 0.3% (left) and 0.5% (right) agar. (**C**) The results obtained using the stab technique in M9 and KB media.

To assess whether these changes in the gene expression generate a motility phenotype in *P. syringae* pv. phaseolicola related to temperature, we evaluated the motility pattern of this bacterium at 18°C and 28°C. To ensure that the bacteria were in the same physiological condition as when the microarray analysis was performed, *P. syringae* pv. phaseolicola NPS3121 cells grown at 18°C and 28°C (OD _600_: 1.1 and 1.2) were inoculated in semisolid M9 media containing 0.3%, 0.4%, and 0.5% agar and incubated at 18°C and 28°C. The results showed that under these conditions the bacterium was not motile despite gene induction at 18°C (Figure 4A). Additionally, motility assays in KB media were performed, using the conditions that have demonstrated motility in related pathovars [41,42]. Plates and glass tubes with semisolid KB media were used to evaluate motility at the mentioned temperatures. Again, the *P. syringae* pv. phaseolicola NPS3121 strain was not motile under these conditions compared to *P. syringae* pv. tomato DC3000 and *P. syringae* pv. tabaci, which showed motility at both temperatures and where it was observed that low temperatures appear to affect their motility (Figures 4B and 4C). This non-motile phenotype of *P. syringae* pv. phaseolicola NPS3121 had been previously reported [41,43], and further experiments are required to determine the conditions in which this bacterium can be motile and to evaluate the effect of low temperature in this process.

### Low temperature induces oxidative stress and iron metabolism

Another group of genes differentially expressed at 18°C correspond to genes related to iron metabolism (Cluster 6). Iron fulfills a vital role in virtually all organisms because of its participation in several cellular processes. Because iron is in short supply in many habitats, bacteria secrete siderophores, compounds that are specific iron chelators, to mobilize inside the cell through membrane receptor molecules [44]. Two genes, PSPPH_3753 that encodes a protein related to siderophore synthesis and PSPPH_1923 that is involved in pyoverdine synthesis (a major siderophore of the fluorescent *Pseudomonas sp*.), were induced at 18°C relative to 28°C [45]. Likewise, the gene encoding sigma factor protein PvdS, which is required for expression of pyoverdine synthesis genes, was induced under these conditions [46]. The induction of this PvdS protein was validated by RT-PCR analysis (Figure 3). One gene encoding the regulatory protein FecR (PSPPH_2117) and proteins involved in iron transport were also included in this group. It is known that in *P. aeruginosa*, the Fur protein is the master regulator of iron homeostasis. It represses pyoverdine synthesis via negative regulation of the *pvdS* gene under high iron concentrations. However, in iron-limiting conditions, Fur repression is released and transcription can occur [47]. It has been reported that PvdS sigmulon is conserved among the fluorescent pseudomonads, including the *P. syringae* group [46]. Although the *fur* gene was not printed on our microarray, the functional status of Fur protein can be inferred as inactive because the genes regulated by this protein are induced in the conditions evaluated. This expression profile simulates conditions of iron deficiency.

To phenotypically evaluate changes in the expression of siderophores synthesis genes in function of temperature, we performed quantitative analyses of siderophores at 18°C and 28°C. The results of these assays showed that at 18°C, the amount of siderophores in the culture supernatant was higher (58.6 ± 0.39 μM) compared to when the bacterium is grown at 28°C (20.53 ± 0.844 μM). Thus, the results demonstrate that low temperature induces siderophores production by the bacterium.

Additionally, it has been reported that in several bacteria, the Fur protein positively regulates the expression of genes involved in various pathways in response to large iron amounts, such as oxidative stress genes (e.g. catalases) [47]. In our microarray, the PSPPH_3274 gene (encoding the catalase KatB) was induced at 18°C, which would be inconsistent with our hypothesis about an inactive status for the Fur protein at low temperatures. However, several reports have indicated an association between decreased environmental temperature and increased intracellular oxidative stress in bacteria. Thus, there is evidence that free radical production (superoxide O_2_^-^, hydrogen peroxide H_2_O_2_, or hydroxyl radical HO^-^) in bacterial cells is stimulated at low temperatures, apparently in an iron-independent manner. Therefore, the expression of oxidative stress adaptation genes, such as catalases, increase considerably [48,49]. A similar response may occur in our strain, justifying the observed induction in the catalase gene, as low temperature induces free radical production in cells, in turn increasing catalase production. The expression of the gene encoding catalase (KatB) was evaluated by RT-PCR analysis (Figure 3). Furthermore, it has been reported that iron-starvation inducible genes are also induced in response to oxidative stress in *P. aeruginosa*. This response appears to be due to a transient loss of Fur repressor function [50]. These observations are consistent with our data and support our hypothesis about the inactive status of the Fur protein at low temperatures.

Additionally, within Cluster 6, we also found PSPPH_1309, which encodes the cysteine desulfurase IscS, and PSPPH_1311, which encodes iron-sulfur cluster assembly protein IscA, both components of ISC (iron-sulfur cluster) system essential in the biogenesis of iron-sulfur (Fe-S) proteins in bacteria. It has been observed that some pathways involved in Fe-S cluster assembly operate under iron starvation and oxidative stress conditions [51,52], which agrees with the results obtained.

On the other hand, several reports have indicated a correlation exists between the uptake-transport iron system and motility process and biofilm formation. Thus, iron deficiency stimulates twitching motility, a form of surface motility that is inconsistent with microcolonies and biofilm formation [53]. This is consistent with the results obtained in our experiments, because iron metabolism genes and siderophores production are induced, simulating iron deficiency conditions, and motility processes appear to be favored, whereas biofilm or extracellular polysaccharide formation is decreased (see data below).

### Hypothetical proteins and proteins with unknown function are induced at 18°C

Among the differentially regulated genes induced at 18°C, we found 15 genes that hypothetically encode conserved proteins (Cluster 7). Additionally, Cluster 8 has genes that could not be grouped into any specific biological process but showed high transcript levels at 18°C relative to 28°C. Within this cluster are genes encoding various transcriptional regulators, a gene that encodes an ATP-dependent helicase, DinG family (PSPPH_1406), and the PSPPH_4151 gene that encodes RNA polymerase sigma-54 factor RpoN whose expression was validated by RT-PCR assays (Figure 3).

### Low temperature represses alginate synthesis in *P. syringae* pv. phaseolicola NPS3121

With regard to repressed genes identified at 18°C, we found a group of genes involved in alginate synthesis (Cluster 9). The genes enconding AlgX (PSPPH_1112), AlgG (PSPPH_1113), AlgE (PSPPH_1114), AlgK (PSPPH_1115), and AlgD (PSPPH_1118), as well as the PSPPH_1119 gene that encodes a hypothetical protein, were included in this cluster. Alginate is an extracellular polysaccharide (EPS) produced by bacteria that is secreted into growth media and involved mainly in biofilm formation. Production of this co-polymer by *P. syringae* and *P. aeruginosa* has been previously reported [54,55]. Alginate production by *P. syringae* has been associated with increased epiphytic fitness, resistance to desiccation and toxic molecules, and the induction of water-soaked lesions on infected leaves. Studies have shown that alginate functions in the virulence of some *P. syringae* strains and facilities the colonization and/or dissemination in plants [55]. Although *P. syringae* pv. phaseolicola virulence is favored by low temperature, alginate production by this strain appears to be repressed under these conditions. RT-PCR analyses confirmed the repression mediated by low temperatures of *algD*, the first gene of the alginate biosynthetic operon (Figure 3). The repression of alginate genes mediated by low temperature also has been observed in *P. syringae* pv. syringae, where the expression of *algD*, was induced at 28°C and significantly lower at 18°C [56]. To validate the microarrays results in *P. syringae* pv. phaseolicola NPS3121, the effect of temperature on EPS production (including alginate) was evaluated. Quantitative analyses showed that at 18°C the production of EPS is lower (76.65 ± 4.09 μg) compared to when the bacterium is grown at 28°C (192.43 ± 14.11 μg). Thus, the results demonstrate that the low temperatures decrease EPS production by the bacterium. Alginate gene regulation is complex and varies between species. In *P. aeruginosa,* it has been reported that sigma factor-54 (RpoN) represses *algD* expression by sigma factor antagonism [57]. A similar phenomenon could be occurring in our strain, because the expression levels of the *rpoN* gene (PSPPH_4151) are consistent with the low expression of alginate genes. Furthermore, it has been reported that a coordinated expression exists between flagellum synthesis and EPS production. In *P. aeruginosa*, the FleQ protein, a master regulator of flagella genes, represses the expression of genes involved in EPS synthesis, leading to planktonic cells. When this repression is released, the flagellum genes are repressed and EPS production is favored [58]. The alginate gene repression observed in our microarray, could also be due to repression exerted by FleQ protein, which is induced in our experiment, in a similar manner to what occurs in *P. aeruginosa.* Thus, the results of the microarray are consistent with the fact that EPS production (e.g., alginate) is decreased at low temperatures whereas expression of motility genes is favored.

### Genes involved in plant-pathogen interactions are affected by low temperature

Another group of genes repressed at 18°C comprise Cluster 10 and correspond to genes related to plant-pathogen interactions (Table 2). Four of these genes encode Type III effector proteins (T3EFs): HopAB1, HopW1, HopD1, and AvrB2, but the other genes are involved in cell wall degrading enzyme synthesis, such as pectin lyase (PSPPH_3992) and polygalacturonase (PSPPH_A0072). The repression of some T3EFs genes and cell wall degrading enzyme genes was validated by RT-PCR (Figure 3). The classification of these genes as pathogenicity and/or virulence factors in *P. syringae* pv. phaseolicola has been previously reported [18]. It known that phytopathogenic bacteria suppress plant innate immunity and promote pathogenesis by injecting directly into host cells effector proteins (T3EFs) by a type III protein secretion system (T3SS). However, in the majority of cases, neither the mode of action nor the targets of these effector proteins within the plant are known [59]. In addition, phytopathogens synthesize and secrete various cell wall degrading enzymes, which facilitate pathogen entry and nutrient release for its growth [1]. Thermoregulation of these genes has been observed in other bacterial phytopathogens, where their expression is favored at low temperatures, a phenomenon opposite to data obtained in our experiments [4]. However, it has been reported that in *P. syringae* pv. tomato DC3000, iron bioavailability regulates the expression of T3SS component genes. Thus, high iron concentrations induced expression of genes such as *hrpRS* and *hrpL*, which in turn regulates the expression of T3SS genes, by an as yet unknown mechanism [60]. Based on this, our microarray results might be explained by the fact that the uptake-transport iron genes were induced, mimicking iron limiting conditions, which could lead to the observed repression of T3SS genes.

### Genes related to the Type IV secretion system (T4SS) are repressed at 18°C

Another group of genes differentially repressed at 18°C comprise Cluster 11. They include genes related to the type IV secretion system (T4SS), which is closely related to systems involved in the conjugal transfer of DNA (Table 2). Nine of these genes encode conjugal transfer proteins and two encode transcriptional regulators, all within plasmid B (pPh1448B) of *P. syringae* pv. phaseolicola (Figure 2) [18]. The pPh1448B plasmid belongs to the well-described pPT23A plasmid family, whose members have been demonstrated to play an important role in the interaction of the *P. syringae* pathogen with host plants [61]. Many of the pPT23A family plasmids are known to be conjugative plasmids. The putative T4SS encoded in the pPh1448B plasmid has been classified as a type IVA system, due to its high similarity with the type IV secretion genes of *Agrobacterium tumefaciens* (the *virB* operon and *virD4*) [61]. In addition to functioning in conjugation, some bacterial T4SSs are capable of delivering effector proteins or toxins to host cells, thus acting as virulence factors [62]. However, in the case of *P. syringae* pv. phaseolicola 1448A T4SS, it has been suggested to have a role in conjugal transfer of DNA rather than virulence-related protein translocation [18]. Thermoregulation of some T4SS genes in various bacteria has already been reported, similar to our results in this study [4]. More experimental work is necessary to elucidate the role of these genes in *P. syringae* pv. phaseolicola NPS3121 and their relationship to temperature.

### Low temperature represses the heat shock response

Another group of genes repressed at 18°C correspond to those encoding heat-shock proteins (Cluster 12). Genes that encode the HslVU and GrpE heat-shock proteins, as well as the genes encoding DnaK, GroEL, and ClpB chaperones were included in this cluster. Heat shock proteins (HSPs) are a class of functionally related proteins that are responsible for monitoring the state of protein folding in cells. They function as molecular chaperones, facilitating the folding of partially or fully unfolded proteins. Their expression is increased when cells are exposed to elevated temperatures or other stresses, to cope with protein damage. If however, the temperature decreases, a reverse response is observed and heat-shock gene transcription decreases [63]. This latter behavior is similar to the results obtained in our experiments, where the low temperature decreased the transcript levels of heat-shock genes. In *E. coli*, HSP synthesis is repressed during growth at low temperatures [64]. A similar response has been observed in *P. putida*, where low temperatures also decrease the expression of these genes [65].

### Transcription and replication are repressed by low temperature

Cluster 13 includes genes involved in nucleic acid synthesis. Two of these genes (PSPPH_4598 and PSPPH_4599) encode RNA polymerase beta subunits involved in mRNA synthesis. Three of these genes (PSPPH_2495, PSPPH_B0043, and PSPPH_A0002) are related to the replicative process of DNA synthesis. This result suggests that both processes are affected by low temperature in *P. syringae* pv. phaseolicola NPS3121, which is consistent with the decreased growth rate observed. This behavior is similar what was previously observed in *P. putida* where low temperature also reduces proteins involved in the transcription and replication processes [65].

Finally, similar to the analysis and clustering of activated genes, repressed genes at 18°C that hypothetically encode conserved proteins were grouped into Cluster 14. Likewise, those genes whose products could not be grouped into any specific biological process were included in Cluster 15. The relationship of these genes to the physiology of the bacterium to low temperatures remains unknown and more experimental work is required.

## Conclusions

In general, the results of the microarray provided us with a global view regarding the physiology of *P. syringae* pv. phaseolicola NPS3121 at 18°C, a temperature that favors disease development and produces the major virulence factor of the bacterium. The results showed that common processes in response to low temperature, such as cell-envelope remodeling, transcription, translation, and the heat-shock response, are also affected in this bacterial phytopathogen. In addition, low temperatures influence phaseolotoxin synthesis as well as the expression of various virulence factors involved in disease development. Furthermore, our data show low temperature-dependent expression of T6SS, thus being the first report about the expression of this cluster of genes in *P. syringae* pv. phaseolicola. In general, the expression profile obtained in this study suggest that low temperatures generate an oxidative stress in the bacterium, which leads to expression of uptake-transport iron genes (simulating iron starvation conditions) that in turn are related to the expression of various processes such as motility, biofilm production, and T3SS. From the data obtained in this study, we can begin to understand the temperature dependent strategies used by this phytopathogen during host interactions and disease development.

## Methods

### Bacterial growth conditions and RNA isolation

The *P. syringae* pv. phaseolicola NPS3121 strain was grown at 18°C and 28°C in M9 minimal media supplemented with 0.8% glucose as the carbon source. The growth conditions were as follows: pre-inoculums (25 mL) of *P. syringae* pv. phaseolicola were grown in M9 minimal media overnight at 28°C. The cells were washed once with M9 media and inoculated into 200 mL of M9 minimal media at optical density (OD _600nm_) 0.1. To evaluate the effect of temperature, the cultures were incubated at 18°C or 28°C and grown until they reached the transition phase (OD_600nm_ 1.1 at 18°C and 1.2 at 28°C) and RNA was extracted. For RNA isolation, the cells were recovered by centrifugation at 10,000 rpm for 10 min at 4°C, washed with sterile deionized water, and stored at −80°C. The supernatants from each culture were removed for phaseolotoxin production assays. Total RNA was extracted using the TRIzol Reagent following the manufacturer´s instructions (Invitrogen, CA, USA). A second purification step was performed using RNeasy MinElute spin columns (Qiagen, CA, USA) to remove any contaminating DNA. RNA was eluted in 50 μL of diethylpirocarbonate (DEPC)-treated water. RNA concentration was determined using a ND-1000 spectrophotometer (NanoDrop). RNA integrity was verified by analytical agarose gel electrophoresis.

### Phaseolotoxin assays

Phaseolotoxin production by *P. syringae* pv. phaseolicola was assayed using the *E. coli* JM103 strain growth inhibition assay as previously described [66]. In each case, plates containing arginine (10 mM) were used as controls to confirm that growth inhibition was due to phaseolotoxin effects.

### Microarray processing, data acquisition, and statistical analyses

Previously, our group constructed a DNA microarray of *P. syringae* pv. phaseolicola NPS3121 (GEO accession number GPL7115) with 1911 probes of an average length of 2.4 kbp, which represents approximately 1X of the *P. syringae* pv. phaseolicola NPS3121 genome. This microarray contains also several PCR products corresponding to various genes with known functions that were printed as controls [67]. To perform this study, we used this *P. syringae* pv. phaseolicola NPS3121 DNA microarray. Each microarray experiment was repeated six times: two technical replicates with the same RNA samples and three biological replicates using RNA isolated from a different culture. cDNA synthesis, labeling, hybridization, washing, and chip scanning were performed at the Microarray Core Facility at CINVESTAV-LANGEBIO. Hybridized microarray slides were scanned (GenePix 4000, Axon Instruments, Inc) at a 10-μm resolution, adjusting the laser and gain parameters to obtain similar levels of fluorescence intensity in both channels. The spot intensities were quantified using Axon GenePrix Pro 6.0 image analysis software. First, an automatic spot finding and quantification option of the software was used. Subsequently, all spots were inspected individually and in some cases, the spot diameters were corrected or the spots were removed from the analyses. The mean of the signals and the median of backgrounds were used for further analyses. Raw data were imported into the R.2.2.1 software. Background signals were subtracted using Robust Multichip Analysis (RMA) whereas the normalization of the signal intensities within slides was carried out using “print-tip loess” method and the LIMMA package. Normalized data were log2 transformed and fitted into mixed model ANOVAs using the mixed procedure. The *p*-values of the low temperature (18°C) effect were adjusted using the False Discovery Rate method (FDR). Differentially expressed genes were identified using cut-off criteria of ≥1.5 for up-regulated and ≤0.6 for down-regulated genes (FDR, p-value ≤ 0.05). Analyses of distribution and the location of differentially expressed genes in the *P. syringae* pv. phaseolicola 1448A sequenced genome were performed using the GenoMap software [68].

### Microarray validation by reverse transcription-PCR analyses

To validate the microarray data, we performed reverse transcription (RT)-PCR analyses. The expression levels of several genes with different biological functions were evaluated by this technique. These experiments involved independent biological experiments from those used for microarray analyses. DNA-free RNA was obtained as described above and the integrity of the RNA was evaluated by agarose gel electrophoresis. Total RNA (200 ng) was used for RT-PCR using the Superscript one-step kit (Invitrogen). Controls used for each set of primers were 1) PCR without the reverse transcription step to verify the absence of DNA, 2) RT-PCR performed without RNA templates to detect any contaminating DNA/RNA, 3) PCR performed using genomic DNA as a template to ensure primer fidelity, and 4) amplification of a portion of the 16S rRNA operon as an internal control of the reaction. The RT reaction was performed at 50°C for 30 min. PCR amplification was performed at 94°C for 2 min for 1 cycle; 94°C for 30 s, 55–58°C for 30 s, and 72°C for 1.0 min for 20–28 cycles; and 72°C for 10 min for 1 cycle .

### Molecular biology techniques

Routine techniques were performed using standard protocols [69]. Genomic DNA of *P. syringae* pv. phaseolicola NPS3121 was isolated as described previously [70]. PCR products were amplified with Platinum supermix (Invitrogen). Primers were designed using Vector NTI Software (Invitrogen), with reference to the previously reported sequence of the 1448A strain (Gene Bank accession no. CP000058) [18]. The oligonucleotide primers used in this study are listed in Additional file 1.

### Motility assays

To evaluate the motility of *P. syringae* pv. phaseolicola NPS3121 and the influence of temperature on this process, three strategies were used. The swimming and swarming motility of *P. syringae* pv. phaseolicola NPS3121 were assessed on semisolid KB plates containing 0.3% and 0.5% agar, respectively, as described in previous studies [41,42]. The cells were grown in KB broth overnight at 28°C, and harvested and resuspended in KB to OD_600_ = 1. 50 μL of bacterial suspensions were inoculated on filter disks (6 mm in diameter) and placed in the center of the plate. Plates were incubated for 24 h at 28°C and 18°C before photography. A second strategy was performed to evaluate the swimming and swarming motility of *P. syringae* pv. phaseolicola NPS3121. To ensure that the bacteria were in the same physiological condition as when the transcriptome analysis was performed, the *P. syringae* pv. phaseolicola NPS3121 strain was grown in M9 media at 28°C and 18°C until they reached the transition phase. Bacterial suspensions (50 μL) were inoculated on filter disks (6 mm in diameter) and placed in the center of semisolid M9 plates containing 0.3%, 0.4%, and 0.5% agar. Plates were incubated for 48 h at 28°C and 18°C. Finally, motility was also evaluated using the stab technique in semisolid KB and M9 media (0.3% and 0.5% agar) in glass tubes. The tubes were incubated at 28°C and 18°C for 48 h. As controls, we used the *P. syringae* strains pv. tomato DC3000 and pv. tabaci PTBR2004. Experiments were performed three times with three replicates per treatment.

### Quantification of siderophores

Siderophore production into the culture supernatant by bacterial strains was determined using chrome azurol S (CAS) liquid assays as previously described [71]. Briefly, the *P. syringae* pv. phaseolicola NPS3121 strain was grown in M9 media at 28°C and 18°C until they reached the transition phase. The supernatant was recovered by centrifugation at 8,000 rpm for 15 min at 4°C and filtered through a 0.45-μm-pore-size filter (Millipore). For siderophore quantification, a standard curve was prepared with desferoxamine mesylate. Experiments were performed three times with four replicates per treatment.

### Quantitative analyses of EPS

The quantification of EPS was performed as previously described with some modifications [72,73]. The *P. syringae* pv. phaseolicola NPS3121 strain was grown in M9 media at 28°C and 18°C until they reached the transition phase [the growth stage in which the microarrays analysis was performed and the repression of EPS synthesis genes (alginate) was observed]. The bacterial cells were harvested by centrifugation at 8,000 rpm for 15 min at 4°C. After centrifugation, the supernatant was mixed with three volumes of ice-cold 95% ethanol (with stirring) for 24 h at −20°C to precipitate the extracellular polysaccharide (EPS). EPS was recovered by centrifugation at 10,000 rpm for 20 min at 4°C. The pellet was washed twice with 95% ethanol and once with absolute ethanol. Quantification of the EPS was performed using the phenol-sulfate method. Total EPS was measured using a glucose standard curve. Experiments were performed three times with four replicates per treatment.

### Microarray data accession

The microarray data from this study is available on the GEO database at http://ncbi.nlm.nih.gov/geo with the accession number GSE38423.

## Competing interests

The authors have declared that no competing interests exist.

## Authors’ contributions

JLA-G and AH-M contributed to experimental design, performed experiments, analyzed data, and drafted the manuscript. JRP-A performed experiments and analyzed data. AA-M conceived the study, contributed to experimental design, and edited the manuscript. All authors read and approved the final manuscript.

## Supplementary Material

Additional file 1 This Word file contains the sequence of oligonucleotides used in the RT-PCR assays. Click here for file
