# Supplementary material for: Transcriptional profile of P. syringae pv. phaseolicola NPS3121 at low temperature: Physiology of phytopathogenic bacteria
Source: BMC Microbiol. 2013 Apr 12;13:81. doi: 10.1186/1471-2180-13-81 (PMC3639832; doi:10.1186/1471-2180-13-81)
Supplement: Additional file 1 — This Word file contains the sequence of oligonucleotides used in the RT-PCR assays. [file 1471-2180-13-81-S1.docx]

**Oligonucleotides used in this study.**

| **Gene/Primers** | **Sequence (5´- 3´)** |
| --- | --- |
| ***argK*** |  |
| L10001 | CTTTGATGGTATGCATGCGGTT |
| L10002 | GGAAGAACTGGCCAAACATTCG |
| ***phtA*** |  |
| 1417 | GAGCGTCACGCTCTATTTTGGTGC |
| Ptx1I4D | CAGATACGAACAACACTACT |
| ***desI*** |  |
| P16881 | TCAACAACATCCACGGGCAT |
| G720 | GATATCGCAGCAACACCCATAAAAC |
| ***phtL*** |  |
| L100050phtLs | GTACGTTCCAGCTGCGCCATTGAAG |
| FIP12718 | TACGTTGCCCGGTGTCGACA |
| ***amtA*** |  |
| BRL519 | TTCATTCAAACCTCGCCCGTGTG |
| BRL520 | TGAAAGGAGCCGCCGAAACTATTG |
|  |  |
| **PSPPH_4547** |  |
| L100140PH4547f | CCAGTTTTTCAACGCGGCGC |
| L100140PH4547r | ACATTGAGGCGCACGGCACA |
| **PSPPH_4553** |  |
| L100142PH4553f | CATCGGGCAAGGATTCTGCT |
| L100143PH4553r | CCGGGATCTCGAATACTGAA |
| **PSPPH_4554** |  |
| L100144PH4554f | GAAAATTGGGAGTTGAATCATG |
| L100145PH4554f | GCATTAACCTTGTATTTGGC |
| **FleQ** |  |
| FleQ 1F | GATCTGGCGGTTATCCTGAA |
| FleQ 1R | TACCACTTCCTTGCCAGTCC |
| **pvdS** |  |
| L100277-1909D | GACTCACCATTACTCCAGGC |
| L100278-1909R | AGACGGTACATCTCGAACGC |
| **katB** |  |
| katB 1F | ATTAAACTGGTCCAGACACATGG |
| katB 1R | CATTTGAATACAGCAGTGTCAGC |
| **Type III effector *virPphA*** |  |
| F069-27 | CGGTCCGCCAACAAAGAAT |
| F069-28 | GAGCCGCCGGGTCAGTTAT |
| **Type III effector *avrPphC*** |  |
| F069-21 | CGGGCGATGCGTCAAAAAT |
| F069-22 | GGCCGCTCAAAAAGGTTAG |
| **Type III effector *avrPphD*** |  |
| F069-23 | ACTGTTGGGTGAGAAGAAT |
| F069-24 | TAAATGCGCAACTGAACTA |
| ***pnlA*** |  |
| L100184PL1 | CTATCGTGATTCCCATGAACCAACA |
| L100185PL2 | ACACCCTTCAACGCTTGACCAGCTT |
| **polygaracturonase** |  |
| L100176poliF | GAGCCTCAGGCACCACGGATTTCAT |
| L100177poliR | TACTGTCACTTTACCCACGCAGGCG |
| ***algD*** |  |
| F069-1 | CCGCGGTCACGATGTAGTT |
| F069-2 | TTCCGCCAGGTCGTCAGTA |
| **rpoN** |  |
| L10007-rpoN | TGAAACGGATCCTAGTCTTGAGAATGGGCCAG |
| L10008-rpoN | GACCAAGGATCCTGCTATCACTCAACGGCTTT |
| **PSPPH_0122 *hcp*** |  |
| L100291PSPPH0122F | TGGCATTTGACGCATACA |
| L100292PSPPH0127R | GGTCCCAGCCGCCAGTG |
| **PSPPH_0127 *hsiJ*** |  |
| L100307PSPPH0127F | GACACCCTGCCTGACCTCAACGAAG |
| L100308PSPPH0127R | CACCAGCGAGGCCGCCGGGTCCTGA |
| **PSPPH_0131 *hsiG*** |  |
| L100311PSPPH0131F | GGACCGACTGCGCTTTTTCCTTGAT |
| L100312PSPPH0131R | CCGCCGCACGCGATCGATAGAGACC |
| **16S ribosomal** |  |
| L10003416sF | ACACCGCCCGTCACACCA |
| L10003416sR | GTTCCCCTACGGCTACCTT |
|  |  |
|  |  |
